# Strategies for Coping with Minority Stress among Queer Young Adults: Usage Frequency, Associations with Demographics, and Mental Health

**DOI:** 10.3390/ijerph21081052

**Published:** 2024-08-10

**Authors:** Yinuo Xu, William J. Hall, McRae Scott, Yutong Gao, Pin-Chen Chiang, Denise Yookong Williams, Ankur Srivastava, Magdelene E. Ramon, Adam R. Englert

**Affiliations:** 1 School of Social Work, University of North Carolina at Chapel Hill, Chapel Hill, NC 27599, USA; wjhall@email.unc.edu (W.J.H.); mcrae_scott@med.unc.edu (M.S.); pcchiang@unc.edu (P.-C.C.); dwilliams3@unc.edu (D.Y.W.); ankursri@unc.edu (A.S.); magdelene_ramon@unc.edu (M.E.R.); arengler@unc.edu (A.R.E.); 2 School of Education, University of North Carolina at Chapel Hill, Chapel Hill, NC 27514, USA; gyutong@unc.edu

**Keywords:** coping strategies, minority stress, queer young adults, anxiety, depression

## Abstract

Queer young adults report significantly higher levels of anxiety and depression than their heterosexual counterparts, which is linked to sexual minority stress. Therefore, it is important to understand the coping strategies employed by this population to navigate minority stress and how coping strategies may impact mental health outcomes. Drawing from a U.S. national diverse sample of 387 queer young adults (ages 18–39 years), we analyzed descriptive results of 11 behavioral strategies to cope with minority stress and used ordered logistic and linear regression to examine the following objectives: the frequency of the use of each coping strategy, and the associations between each strategy and demographic characteristics as well as depression and anxiety. Results revealed that avoidance and talking with friends were the most frequently utilized coping strategies, while prayer/religious activities and counseling/psychotherapy/support groups were infrequently used. We examined utilization preferences of coping strategies across demographic factors (e.g., assigned sex at birth and sexual orientation). The use of counseling/psychotherapy/support group was positively associated with mental health symptoms, while exercise and mindfulness/mediation were associated with lower mental health symptoms. Our findings provide insights for mental health researchers and professionals in selecting appropriate coping strategies for queer young adults in prevention and intervention efforts.

## 1. Introduction

Mental health disparities between queer (we use queer as an umbrella term to refer to people who are not heterosexual, which includes but is different from people with queer-identifying sexual orientation, i.e., lesbian, gay, bisexual, pansexual, queer, and other sexual minority identities) young adults and their heterosexual counterparts are significant. Results from the 2021 and 2022 National Surveys on Drug Use and Health show that lesbian, gay, and bisexual adults are more likely than heterosexual adults to experience mental health problems, including major depressive episodes, serious thoughts of suicide, and suicidal planning [1]. Results from another national representative study shows that queer adults experience higher prevalence of major depression and generalized anxiety disorder than heterosexual individuals [2]. A queer identity itself does not cause queer-related mental health disparities. Rather, according to minority stress theory (MST) [3], sexual orientation disparities in mental health occur because queer people experience socially based stressors called sexual minority stress, due to their minoritized identities, which are in addition to general life stressors (e.g., injury, financial challenges, interpersonal challenges) facing individuals generally. Sexual minority stressors are related to the stigma and prejudice present in heterosexist cultures [3,4].

Despite the presence of sexual minority stressors, MST also suggests that queer individuals may use diverse coping mechanisms in response to minority stress [3], and these coping strategies may change the influences of minority stress on mental health [5]. Coping strategies are responses intended to reduce the physical, emotional, and psychological burden of stressful events [6]. The appraisal theory of coping posits that individuals select strategies based on their assessment of stressors [7]. The evaluation and use of these strategies, though intended to benefit well-being, may consequently prove adaptive or maladaptive depending on their outcomes [6]. It would be helpful to know which coping strategies are commonly and infrequently used by queer young adults. Studies on coping strategies in queer populations have revealed diverse patterns, with common strategies including seeking social support from the queer community, self-concealment, and emotional numbing [8,9]. However, there is still a lack of research that assesses and compares the frequency of various coping strategies in a large, national study of queer young adults, which could inform interventions aimed at improving well-being and reducing stress in this population. Furthermore, queer young adults are not a homogeneous group, yet research on this population has often focused on White, highly educated, affluent individuals [10]. This narrow focus may fail to capture important subgroup differences in coping strategies. Recent studies have begun to provide demographic details on coping strategies in queer populations, mostly the coping style preferences based on sexual orientation [11,12], age, and race/ethnicity [8]. For example, older Black queer individuals may tend to use disengaged coping strategies, and adaptive coping strategies (e.g., help seeking and trying new activities) were common among queer women. However, most studies have not quantitatively tested whether and how demographic characteristics are associated with different coping strategies. This gap in research limits our understanding of intersectional factors that may influence coping mechanisms, potentially hindering the development of targeted interventions for diverse queer young adults.

In the face of minority stress, some studies have examined how coping strategies are related to mental health outcomes among queer young adults. Helpful coping strategies included meditation and mindfulness [13,14,15], religious-related coping or spiritual activities [16,17,18], support from family or friends [19,20,21,22], writing or journaling [23,24], and engaging in queer-affirming media [25]. On the other hand, avoidance and ignoring strategies were associated with maladjustment and poorer mental health outcomes [11,26]. Moreover, evidence for the role of activism as coping has been mixed [27,28]. A handful of other studies have examined the benefits of other general coping strategies to reduce anxiety and depression among queer people, yet not coping specifically with minority stress, such as exercise and physical activity [29] and queer-affirmative cognitive behavioral therapy [30,31,32].

### Current Study

Given that most studies on queer coping have only examined the use of and the mental health benefits or consequences of one or a few coping strategies, contemporary evidence is needed to examine a wider range of coping strategies used by queer individuals to manage minority stress. With the exploration of how different coping strategies relate to depression and anxiety, such evidence can inform evidence-based interventions and mental health services for queer populations. This study aims to explore and compare how strategies to cope with minority stressors is related to the mental health outcomes (anxiety and depression) among queer young adults. The research questions are: (1) What is the frequency of use of strategies for coping with discrimination/oppression among queer young adults in the United States? (2) How are demographic characteristics related to engagement in various coping strategies? (3) What are the associations between various coping strategies and symptoms of depression and anxiety among queer young adults? Due to insufficient prior research on this topic with this population, we did not develop hypotheses regarding the frequency of different coping strategies or their demographic correlates. These two research questions were more exploratory in nature. Nonetheless, for the third research question, we hypothesized that avoidance and ignoring would be associated with poor mental health outcomes, given results in prior studies [11,26]. We also hypothesized that strategies previously found beneficial for queer adults’ well-being, such as meditation, mindfulness [13,14,15], and support from family and friends [19,20,21,22], would be associated with lower rates of anxiety and depression.

## 2. Methods

### 2.1. Sample Recruitment and Procedures

Data for this study were derived from an initial wave of an online quantitative survey. A sample of queer adults was recruited through CloudResearch (formerly TurkPrime), a participant-sourcing platform for online research [33]. Research shows that CloudResearch participants are diverse, have demographic characteristics that approximate nationally representative U.S. samples, and are more representative than in-person or Internet-based convenience samples [34,35,36,37,38,39,40,41]. An online survey was administered in early November 2020 and participants responded to demographic questions, followed by stressor items, and then mental health-related questions. Each participant was paid $10 for completing the survey, which is standard for CloudResearch participants. Participants were included if they identified as a sexual minority (e.g., gay, lesbian, bisexual, queer), were at least 18 years old, lived in the United States, and could read and understand English. Two participation validity check items were embedded in the survey (e.g., “Please select the ‘strongly agree’ response for this item”) to identify participants who potentially did not read the survey items and may have responded indiscriminately. Any participant who failed one of these items was excluded (*n* = 31). In addition, participants who only completed a small part of the initial survey items then stopped were excluded (*n* = 81).

### 2.2. Variables and Measures

#### 2.2.1. Demographics

The survey measured the demographics of age, sex assigned at birth, gender identity, race/ethnicity, and sexual orientation. Response options for gender identity were cis-man, cis-woman, transgender, transgender man or transmasculine, transgender woman or transfeminine, non-binary, genderqueer, gender non-conforming, genderfluid, bigender, polygender, pangender, two-spirit, agender, questioning, and another identity (wrote in by the participant). We categorized participants’ gender identity into cisgender or transgender/gender diverse (TGD). The response options for sexual orientation included: heterosexual or straight, gay or lesbian, bisexual, pansexual, queer, asexual, questioning, and other (write in by the participant). No participants identified as heterosexual or asexual. We then categorized participants’ sexual orientation to gay or lesbian, bisexual and/or pansexual, queer/other (i.e., if participant selected queer, questioning, or other).

#### 2.2.2. Strategies to Cope with Minority Stress

Given the lack of established measures to capture a range of coping strategies for minority stress, we used an 11-item self-report inventory developed by 3 members of the research team who have expertise in behavioral measurement. Participants answered the question, “What strategies do you use to cope with discrimination or social oppression?” by rating their use of 11 domains: meditation or mindfulness; prayer, religious or spiritual activities; activism; counseling, psychotherapy, or support group; exercise or physical activity; talking with friends; talking with family; journaling; reading; creative or artistic pursuits; ignore or avoid. Response options included “Never”, “Sometimes”, or “Often” (coded as 0, 1, and 2).

#### 2.2.3. Mental Health Outcomes

Anxiety. General anxiety symptoms in the past 30 days were assessed by the 21-item Beck Anxiety Inventory [42]. Example symptoms included “Unable to relax” and “Nervous”. Each item was rated on a scale of 0 = “Not at all” to 3 = “Extremely”, higher scores signify higher frequency and severity of anxiety symptoms. According to Beck et al. [42], a composite score of 0–7 corresponds to minimal anxiety levels, 8–15 corresponds to mild anxiety, 16–25 corresponds to moderate anxiety, and 26–63 corresponds to severe anxiety. An overall anxiety symptom composite score was obtained by averaging the responses for all items. In this study, the scale had a Cronbach’s *α* of 0.95, indicating high internal consistency reliability.

Depression. Depressive symptoms in the past 2 weeks were assessed by the 16-item Clinically Useful Depression Outcome Scale [43]. Example symptoms included “I felt sad or depressed” and “I was not interested in my usual activities.” Each item was rated on a scale of 0 = “Never” to 4 = “Almost every day”, higher scores signify higher frequency and severity of depression. According to Zimmerman et al. [43], scores of 0 to 10 correspond to non-depressed; scores of 11 to 20 correspond to minimally depressed; scores of 21 to 30 correspond to mildly depressed; scores of 31 to 45 correspond to moderately depressed; and scores of 46 or higher correspond to severely depressed. An overall depressive symptom composite score was obtained by averaging the scores for all items. In this study, the scale had a high internal consistency reliability with Cronbach’s *α* of 0.94.

#### 2.2.4. General Stress and Minority Stress

We measured participants’ levels of general life stress and sexual minority stress. For general life stress, we used the 41-item Survey of Recent Life Experiences [44], which asks about occurrences of past year stressful life events across 6 domains: social and cultural difficulties (e.g., conflicts with family), work or school challenges (e.g., disliking work or school), time pressures (e.g., not having enough free time), financial problems (e.g., financial burdens), social acceptability problems (e.g., being ignored), and social victimization (e.g., being taken for granted). Each item was rated on a scale of 0 = “Never”, 1 = “Rarely”, and 2 = “Sometimes”. Overall levels of general life stress were obtained by averaging the scores of each item, and a higher score signified higher general life stress. In this study, the scale had a Cronbach’s *α* of 0.94.

Levels of sexual minority stress were measured with the 35-item LGB Multidimensional Stigma Inventory [45], a 7-factor scale that asks about participants’ experiences of sexual minority stress in terms of structural stigma (e.g., lack of support from federal government), intra-community stigma (e.g., not welcomed by the queer community), perceived stigma (e.g., felt excluded from society because of sexual orientation), identity concealment (e.g., pretending to be heterosexual), internalized stigma (e.g., would be happier if being heterosexual), enacted stigma (e.g., been physically assaulted because of sexual orientation), and rejection/harassment (e.g., been called a derogatory name). Each item was rated on a Likert scale of 0 = “Strongly disagree” to “6 = “Strongly agree”. In this study, the scale had high internal consistency reliability (Cronbach’s *α* = 0.90).

### 2.3. Analysis Plan

SPSS (version 28) and Stata (version 18) were used for data management, descriptive statistics, bivariate analyses, ordered logistic models, and multiple linear regression models. There was no more than 1% of missing data for all of the variables of interest. To assess the nature of the missing data, Little’s Missing Completely at Random (MCAR) test was applied. The result was insignificant, suggesting the missing data were missing completely at random. Due to the limited amount of missing data, we employed the expectation maximization (EM) algorithm [46,47]. Prior to linear regression analysis, several diagnostics were performed to examine multicollinearity, influential outliers, heteroscedasticity, residual distributions, and linearity between independent and dependent variables. No multicollinearity problems were found (VIF < 10). No influential outliers or significant heteroscedasticity issues were found. Residuals showed nearly normal distributions for the dependent variables. Plots showed no clear departures from linearity. Variables were approximately normally distributed. A power analysis was conducted using G*Power (version 3.1.9.7). Results indicated that the required sample size to achieve 80% power for detecting a small effect size (*f*^2^ = 0.05) at a significance level of alpha = 0.05 with 11 primary independent variables of interest (i.e., coping strategies) and 12 covariates was *N* = 347 for multiple linear regression (fixed model, *R*^2^ increase). Therefore, our analytic sample size of *N =* 387 was sufficient for the linear regression models. Due to the lack of established standards for estimating power in ordered logistic regression, logistic regression’s power analysis in G*Power was used as a reference tool for sample size estimation. The parameters were set for a one-tailed test with an odds ratio of 1.5, a probability of 0.3, an alpha level of 0.05, and a power of 0.8, which resulted in an estimated sample size of 190. Additionally, based on conventional recommendations [48,49], our sample size of 387 was adequate for logistic regression with 12 independent variables.

## 3. Results

### 3.1. Sample Characteristics

Table 1 presents the demographics for the sample and the descriptive results of variables of interest. Participants for this study included 387 queer young adults aged 18–39 years (*M* = 29.31, *SD* = 5.38). Most respondents identified as White (60.7%), followed by Black/African American (14.2%), Hispanic/Latinx (12.4%), Asian (7.2%), Native Americans (2.8%), and Multi-racial/Multi-ethnic (2.6%). For sex assigned at birth, 62.5% of the respondents reported female and 37.5% reported male. For gender identity, 84.2% identified as cisgender and 15.8% as transgender and gender diverse (TGD; e.g., transgender, transmasculine or transfeminine, non-binary, genderqueer). For sexual orientation, 27.1% identified as gay and/or lesbian, 62.3% as bisexual and/or pansexual, and 10.6% as queer/other.

On average, the participants reported moderate levels of general stress (*M* = 1.31, *SD* = 0.49) and moderate levels of sexual minority stress (*M* = 2.09, *SD* = 0.79). Regarding mental health outcomes, the mean score for anxiety symptoms was 0.82 (*SD* = 0.64). Using the suggested cut-off score (Beck et al., 1988), 25.6% of the respondents reported severe anxiety symptoms, and 23.2% of the respondents reported moderate anxiety symptoms. The mean score for depressive symptoms was 1.22 (*SD* = 0.89). Using the suggested cut-off score (Zimmerman et al., 2008), 23.9% of the respondents reported moderate to severe levels of depressive symptoms, 18.1% of the respondents reported mild depressive symptoms, and 58.0% reported minimal or no depressive symptoms.

### 3.2. Usage of Strategies to Cope with Minority Stress

Table 2 presents the frequency of coping strategies. Ignore or avoid and talking with friends were the most frequently used coping strategies. Prayer or religious/spiritual activities, counseling, psychotherapy, or support group, and journaling were the most infrequently used coping strategies. Creative or artistic pursuits, reading, and exercises or physical activities were moderately used.

### 3.3. Demographics Differences in Strategies to Cope

We used ordered logistic regression to examine how demographic characteristics were related to the usage of each coping strategy, as detailed in Table 3. Individuals assigned female at birth (AFAB) had higher ordered log-odds of using journaling to cope with minority stress, compared to those assigned male at birth (AMAB; OR = 2.11, *p* < 0.01). Compared to cisgender participants, TGD participants had lower log-odds of talking to family to cope (OR = 0.66, *p* < 0.05). For sexual orientation, those who identified as queer/other had higher log-odds of using activism to cope compared to gay/lesbian individuals (OR = 3.17, *p* < 0.01). Compared to White individuals, Black/African American individuals had higher log-odds of utilizing coping strategies of meditation and mindfulness (OR = 2.18, *p* < 0.05), prayer, religious, or spiritual activities (OR = 4.20, *p* < 0.001), exercise or physical activities (OR = 2.47, *p* < 0.01), and talking with family (OR = 2.93, *p* < 0.01). Compared to White individuals, Latinx individuals had higher log-odds of talking with family to cope (OR = 2.11, *p* < 0.05).

Participants experiencing high levels of general life stress showed higher log-odds of utilizing meditation and mindfulness (OR = 1.75, *p* < 0.05), activism (OR = 1.67, *p* < 0.05), counseling, psychotherapy, or support groups (OR = 1.79, *p* < 0.05), creative and artistic pursuits (OR = 1.80, *p* < 0.05), and avoiding or ignoring to cope with minority stress (OR = 2.28, *p* < 0.01). High levels of sexual minority stress were associated with higher log-odds of using exercises or physical activities (OR = 1.46, *p* < 0.01), journaling (OR = 1.75, *p* < 0.001), reading (OR = 2.46, *p* < 0.001), and creative or artistic pursuits to cope (OR = 1.50, *p* < 0.01).

### 3.4. Strategies to Cope and Mental Health Outcomes

Table 4 displays the linear regression results for predicting anxiety and depressive symptoms. The overall model for anxiety was significant (*F*_(23, 363)_ = 13.63, *p* < 0.001) and accounted a large amount of the variance in anxiety symptoms (*R* ^2^ = 0.46, adj. *R*^2^ *=* 0.43). A one-unit increase in the use of counseling, psychotherapy, or support group corresponded to a 0.15 increase in anxiety symptoms (*p* < 0.001). Exercise was significantly and negatively associated with anxiety symptoms, with a one-unit increase in the use of exercise corresponding to a 0.10 decrease in anxiety symptoms (*p* < 0.05). The overall model for depressive symptoms was significant (*F*_(23, 363)_ = 18.82, *p* < 0.001) and accounted a large amount of the variance in anxiety symptoms (*R* ^2^ = 0.54, adj. *R*^2^ *=* 0.51). A one-unit increase in the use of counseling, psychotherapy, or support group corresponded to a 0.09 increase in depressive symptoms (*p* < 0.05). Mindfulness and exercise were significantly and negatively associated with depressive symptoms. A one-unit increase in the use of meditation or mindfulness corresponded to a 0.11 decrease in depressive symptoms (*p* < 0.05), and a one-unit increase in the use of exercise corresponded to a 0.11 decrease in depressive symptoms (*p* < 0.05).

## 4. Discussion

The current study examined the strategies employed by queer young adults to cope with minority stress, their associations with demographic factors, and relationships with anxiety and depressive symptoms.

### 4.1. Use of Strategies to Cope with Minority Stress

Our findings align with previous studies in that social support and avoidance behaviors (e.g., concealing identity and disengaging from discrimination) are common strategies among queer young adults to deal with minority stress [9,50]. Support from friends, especially those within the queer community, is beneficial for queer individuals because these friends can provide empathic listening, relate to their experiences, help navigate identity exploration, and assist in overcoming queer-specific challenges [9,51]. These supportive interactions can foster a sense of social safety [52], which is a crucial protective process regardless of the presence of minority stress. The existing literature indicates that avoiding and ignoring coping strategies are often used in response to minority stressors such as microaggressions and internalized stigma [26,53,54]. This is because such minority stressors can lead to internal conflicts regarding one’s queer identity, which may restrict access to social support systems and result in the use of more avoidant coping strategies [55].

Our study found that prayer or religious activities were among the least frequently used coping strategies, which possibly can be explained by the fact that many ideologies within dominant organized religions involve anti-queer beliefs and stances [56]. Researchers have found that many queer people face dilemmas arising from holding a queer and a religious identity (especially involving anti-queer religions), which could contribute to reduced engagement with religious practices or disaffiliation from religions groups [56]. Recent evidence indicates a trend that younger birth cohorts (e.g., Millennials) are less religious than older cohorts (e.g., Generation X) [57], and that queer individuals are more likely to disaffiliate during emerging adulthood than their heterosexual counterparts [56]. Coping through counseling, psychotherapy, or support groups was another less frequently used strategy. However, around 35% of participants reported ever using this approach, a percentage that closely aligns with Dunbar and colleagues’ findings on mental health service use among queer college students [58]. Despite this utilization rate, there remains a significant unmet mental health service need of queer individuals, which is related to both the lack of understanding of the necessity for mental health support and systemic barriers and negative experiences (e.g., discrimination, non-affirming care) in psychotherapy and other mental health care settings [59,60].

### 4.2. Demographic Characteristics and Strategies to Cope with Minority Stress

Our findings suggest that AFAB queer young adults were more likely to cope through journaling than AMAB individuals. This aligns with previous research on sex differences in coping styles, where females are typically more inclined to adopt emotional or expressive coping styles compared to males [61,62,63].

It is likely that AMAB queer individuals engage in less journaling because they perceive it conflicts with the dominant masculinity ideology, which allows females more than males to express their emotions [64]. Our findings also suggest that compared with cisgender queer individuals, TGD are less likely to cope by talking to family members. This finding is in line with the extant literature on family rejection experiences among TGD individuals when they disclose their gender identity or go through the gender transition process [65,66,67].

Queer/other individuals more often used activism to cope with minority stress, which is consistent with findings from another study [68]. This tendency may stem from queer/other individuals as having more politicized identities (compare to LGB identities), as well as the empowering nature of activism, which has been shown to enhance self-esteem and empowerment, especially among queer-identifying individuals [68,69]. Furthermore, given that queer is viewed as both an empowering and stigmatized identity [70,71], it is likely that queer-identifying individuals are more prone to adopt proactive and progressive strategies like activism in response to minority stress. This warrants further investigation in future research.

Our findings support extant research around Black/African American coping with stress as some studies suggest that social support and spiritual well-being are sources of strength and recovery from hardship among Black/African Americans [72]. Black churches are often cited as an integral part of the community, especially related to coping with hardship and enhancing self-esteem [73,74]. Mindfulness-based strategies are recognized for augmenting spiritual engagement among Black Christians [75,76]. As one study highlights, participants referred to prayer as “speaking to God” and mindfulness as “hearing God” [77], suggesting that meditation and mindfulness strategies may augment or complement religious and spiritual activities among Black/African Americans. Furthermore, the mental health protective role of inclusive faith communities for queer Black individuals, especially support from communities that allow one to reconcile and integrate their queer identities, has also been noted in the previous literature [78,79]. Exercise is another beneficial stress management strategy for the Black community, with studies showing that Black young people engage in more physical activity than their White counterparts [80] and use exercise to manage negative emotions [81]. Given the limited research on how Black queer young adults use mindfulness, spirituality, social support, and exercise to cope with minority stress, future studies should continue to explore the reasons behind their usage and the impact of these strategies on Black queer individuals’ well-being. Our findings also support other research whereby Latinx individuals identified strong social networks and family support as a coping strategy for general stressors or minority stressors. For Latinx adults, support often lessens the likelihood of acculturative stress on mental health issues, such as depression [82], possibly through cultural values such as familisimo [83] or the value for one’s family, emphasizing harmony, warmth, and interconnectedness [84].

Interestingly, our findings on the relationship between minority stress and coping strategies were consistent with the scant literature where higher levels of minority stress were found to be associated with higher odds of solitary coping [85], including exercise, journaling, reading, and creative or artistic pursuits. Studies have found engaging in solitary coping strategies among queer people (e.g., journaling, reading, and expressive writing) help in healing from the experiences of heterosexism and build critical consciousness through shifting perspectives [85,86]. Practices and interventions can take into account the stress levels experienced by targeted queer population and promote community building and social support for those with higher levels of sexual minority stress.

### 4.3. Coping Strategies and Mental Health Outcomes

The use of exercise or physical activity to cope with minority stress was associated with lower anxiety and depressive symptoms in queer young adults, which aligns with the existing literature suggesting that physical activity can be an effective stress reliever that not only improves physical health but also enhances psychological well-being [87,88], possible through the process of modulating physiological stress responses [89]. For queer adults, physical activity can also offer a temporary escape from the stigma and discrimination of daily life, thereby reducing psychological distress associated with sexual minority stress [29]. Future research could explore the roles of queer organized sports and physical activities in mental health and social well-being.

Our findings that the use of mindfulness and meditation were associated with lower levels of depressive and anxiety symptoms supports previous research indicating the mental health benefits of utilizing mindfulness and meditation to cope with minority stress [13,14,15]. Aligning with theoretical perspectives [90], mindfulness and meditation can alleviate symptoms of anxiety and depression in queer young adults by enhancing awareness to the present-moment, improving emotional regulation, and reducing rumination. In a qualitative study, it was observed that individuals who demonstrated nonjudgmental attitudes, a facet of mindfulness, displayed increased acceptance of their sexual identities and demonstrated greater resilience against external homophobic judgment [14]. Those who integrated mindful actions into their daily life were better equipped to identify positive aspects of their situations, even during challenging circumstances. Consequently, participants who embraced mindfulness during periods of minority stress often reported overcoming obstacles, developing resilience, fostering meaningful connections, and experiencing enhanced mental well-being [14].

Our research findings indicate the use of counseling, psychotherapy, or support groups were associated with higher levels of anxiety and depressive symptoms among queer young adults. This result may initially appear counterintuitive, as these psychotherapeutic interventions are generally designed to alleviate symptoms. One potential explanation is the concept of “getting worse before getting better” in psychotherapy. Engaging in therapy can initially intensify distress as individuals confront and process painful emotions, traumas, and experiences related to minority stress and discrimination [91,92]. This process might temporarily elevate reported symptoms of anxiety and depression, reflecting the challenging nature of addressing deep-seated issues. Another consideration is that queer young adults may seek mental health services only when they experience high levels of distress and mental health issues [91]. For example, McDermott et al. found that queer young people seek mental health support when they reached a crisis point [92], partly because queer individuals often experience stigmatization in healthcare settings, which reduces their motivation to seek care and the likelihood of receiving adequate treatment [93]. It is also possible that some counseling and support groups do not adequately address or adapt to the specific needs of the queer community by effectively mitigating the impact of minority stress [3,94]. In some cases, if not properly managed, services can inadvertently reinforce feelings of isolation or stigma [91]. While our study found a positive association between the use of counseling, psychotherapy, or support groups and higher levels of anxiety and depression in queer young adults, this should not discourage the use of these resources. Instead, it highlights the need for tailored approaches that are informed by an understanding of minority stress and the specific needs of the queer community.

### 4.4. Limitations

One key limitation is the cross-sectional nature of the data, which restricts our ability to discern temporal and causal relationships. Additionally, our assessment of coping strategies was limited to those explicitly measured in the study. Qualitative insights, however, suggest the existence of other prevalent coping mechanisms to deal with minority stress, such as music and social media (e.g., the use of TikTok, YouTube), which were not explored in this research. We did not examine the role of queer-specific coping strategies (e.g., support from queer community), which may be helpful.

### 4.5. Future Directions

Acknowledging the intersectionality within the queer community is essential. Tailored interventions addressing the distinct needs and preferences of racial/ethnic and gender-based groups within the queer population are crucial, given the compounded layers of stress and unique forms of discrimination groups face [8,95]. Therefore, it is imperative for future research to integrate intersectional perspectives that acknowledge and incorporate multiple-minoritized identities and experiences within the queer community. Future studies should also incorporate open-ended questions or adopt a mixed methods design to not only quantify the effectiveness of each queer specific and general coping strategy but also capture other significant ways of coping used in the face of minority stress. Lastly, longitudinal studies are needed to investigate the effectiveness of various coping methods and strategies across different dimensions such as gender identity, race/ethnicity, and sexual identity, as well as to examine the impact of diverse coping strategies on positive identity development and well-being over time.

### 4.6. Practical Implications

Promoting physical activity and mindfulness should be considered as part of a holistic approach to addressing the effects of minority stress and improving mental well-being. For queer individuals, participating in queer-specific sports and physical activities can offer a supportive and inclusive environment that traditional sports often lack. Activities such as queer kickball groups, running groups, and softball teams may provide a space free from the toxic masculinity and rigid gender norms prevalent in many mainstream sports. Additionally, mindfulness and meditation offer valuable tools for managing emotional regulation and reducing rumination, which are linked to better mental health outcomes in queer populations [96,97]. These practices can serve as beneficial spiritual alternatives for queer people who are non-religious or have had negative religious experiences. Our findings also have implications on the importance of enhancing access to LGBTQ+-affirmative psychotherapy and support groups. These services should be well-informed about minority stress and its impact on mental health, ensuring that they do not inadvertently exacerbate feelings of isolation or stigma [98]. Nevertheless, for prevention and intervention practices, a comprehensive approach that combines individual-level coping skills with systemic changes that aim at fostering social acceptance and equity is needed for enhancing the resilience, self-esteem, and overall well-being of queer individuals.

## 5. Conclusions

While a growing body of literature has begun to examine strategies to cope with minority stress among queer individuals, our study stands as one of the first to compare the use of different coping strategies and their associations with demographic factors. This comparative approach provides valuable insights into the gaps in coping strategy utilization and the feasibility of various interventions targeting subgroups of the queer community. Our findings challenge the assumption that commonly viewed adaptive coping strategies are all associated with lower levels of depressive and anxiety symptoms. Physical exercise and mindfulness meditation emerge as the most robust protective factors for mental health outcomes in our study. These results offer valuable guidance for developing targeted interventions and services aimed at enhancing well-being and alleviating stress within the queer community. Our findings regarding the associations between psychotherapy and mental health suggest a need for further investigation into how professional mental health services can be more effectively tailored to the needs of the queer young adult population. Future research should explore ways to optimize these interventions to better address the unique needs of queer individuals.

## Figures and Tables

**Table 1:** Descriptives results of study variables.

Variable	*n*	%
Age (*M*, *SD*)	29.31	5.38
Race/Ethnicity		
White	235	60.7
Black/African American	55	14.2
Hispanic/Latinx	48	12.4
Asian	28	7.2
Native Americans	11	2.8
Multi-racial/Multi-ethnic	10	2.6
Sex Assigned at Birth		
Female	242	62.5
Male	145	37.5
Gender Identity		
Cisgender	326	84.2
Transgender/Gender Diverse	61	15.8
Sexual Orientation		
Gay/Lesbian	105	27.1
Bisexual/Pansexual	241	62.3
Queer/Other	41	10.6
General Life Stress (*M*, *SD)*	1.31	0.49
Sexual Minority Stress (*M*, *SD)*	2.09	0.79
Anxiety Symptoms (*M*, *SD)*	0.82	0.64
Depressive Symptoms (*M*, *SD)*	1.22	0.89

**Table 2:** Usage frequency of coping strategies.

Coping Strategy	Usage Frequency		
	Never (%)	Sometimes (%)	Often (%)
Meditation of mindfulness	185 (47.8)	157 (40.6)	45 (11.6)
Prayer or religious/spiritual activities	306 (79.1)	55 (14.2)	26 (6.7)
Activism	200 (51.8)	143 (37.0)	43 (11.1)
Counseling, psychotherapy, or support group	249 (64.3)	113 (29.2)	25 (6.5)
Exercises or physical activities	131 (34.0)	170 (44.2)	84 (21.8)
Talking with friends	51 (13.2)	188 (48.6)	148 (38.2)
Talking with family	194 (50.3)	156 (40.4)	36 (9.3)
Journaling	235 (61.0)	110 (28.6)	40 (10.4)
Reading	104 (26.9)	176 (45.5)	107 (27.6)
Creative or artistic pursuits	117 (30.2)	148 (38.2)	122 (31.5)
Ignore or avoid	64 (16.6)	165 (42.7)	157 (40.7)

**Table 3:** Ordered logistic regression predicting strategies to cope with discrimination and oppression.

**Independent Variable**	**Meditation or Mindfulness**			**Prayer or Religious/Spiritual Activities**			**Activism**			**Counseling, Psychotherapy, or Support Group**		
	**Coefficient** **(*SE*)**	***OR***	**[95% CI]**	**Coefficient** **(*SE*)**	***OR***	**[95% CI]**	**Coefficient** **(*SE*)**	***OR***	**[95% CI]**	**Coefficient** **(*SE*)**	***OR***	**[95% CI]**
Age	0.00 (0.02)	1.00	[0.97, 1.04]	0.03 (0.03)	1.03	[0.98, 1.08]	−0.01 (0.19)	0.99	[0.95, 1.02]	−0.01 (0.02)	0.99	[0.95, 1.03]
Sex	−0.12 (0.23)	0.89	[0.56, 1.40]	−0.22 (0.29)	0.80	[0.45, 1.43]	0.19 (0.24)	1.21	[0.76, 1.92]	0.20 (0.25)	1.24	[0.75, 2.05]
Gender identity	0.26 (0.29)	1.30	[0.74, 2.28]	0.22 (0.36)	1.25	[0.62, 2.51]	0.12 (0.29)	1.13	[0.64, 2.00]	−0.00 (0.30)	1.00	[0.55, 1.82]
Sexual orientation												
Bisexual/Pansexual	0.18 (0.24)	1.20	[0.74, 1.94]	−0.21 (0.31)	0.81	[0.44, 1.49]	0.13 (0.26)	1.14	[0.69, 1.88]	−0.13 (0.27)	0.88	[0.51, 1.50]
Queer/Other	0.24 (0.38)	1.27	[0.60, 2.70]	−0.48 (0.54)	0.62	[0.21, 1.79]	1.15 (0.38) **	3.17	[1.50, 6.73]	0.72 (0.39)	2.05	[0.96, 4.39]
Race/Ethnicity												
Asian	−0.21 (0.41)	0.81	[0.36, 1.82]	0.53 (0.49)	1.70	[0.65, 4.44]	−0.25 (0.39)	0.78	[0.36, 1.68]	0.10 (0.42)	1.10	[0.48, 2.52]
Black	0.78 (0.31) *	2.18	[1.19, 3.99]	1.44 (0.35) ***	4.20	[2.13, 8.29]	−0.07 (0.31)	0.93	[0.51, 1.70]	0.07 (0.33)	1.07	[0.56, 2.03]
Latine	0.23 (0.31)	1.26	[0.68, 2.32]	0.61 (0.40)	1.83	[0.84, 4.00]	−0.50 (0.34)	0.61	[0.31, 1.18]	−0.02 (0.34)	0.98	[0.50, 1.93]
Multi-racial	0.78 (0.72)	2.19	[0.53, 9.00]	0.28 (0.82)	1.33	[0.27, 6.60]	−0.25 (0.69)	0.78	[0.20, 2.99]	−0.75 (0.81)	0.47	[0.10, 2.30]
Native American	0.58 (0.56)	1.79	[0.59, 5.39]	0.39 (0.82)	1.48	[0.30, 7.38]	−0.88 (0.63)	0.41	[0.12, 1.42]	0.46 (0.57)	1.58	[0.51, 4.88]
General life stress	0.56 (0.24) *	1.75	[1.09, 2.80]	0.42 (0.32)	1.52	[0.81, 2.83]	0.51 (0.25) *	1.67	[1.03, 2.71]	0.58 (0.26) *	1.79	[1.07, 3.01]
Sexual minority stress	0.22 (0.14)	1.25	[0.94, 1.66]	0.27 (0.19)	1.31	[0.92, 1.87]	0.23 (0.15)	1.26	[0.94, 1.67]	0.28 (0.16)	1.33	[0.97, 1.81]
Log likelihood	−359.37			−230.97			−358.38			−303.17		
Likelihood ratio *χ*^2^_(12)_	31.29			36.82			32.89			28.45		
Probability > *χ*^2^	0.002			0.000			0.001			0.005		
Pseudo *R*^2^	0.042			0.074			0.044			0.045		
**Independent Variable**	**Exercises or Physical Activities**			**Talking with Friends**			**Talking with Family**			**Journaling**		
	**Coefficient** **(*SE*)**	***OR***	**[95% CI]**	**Coefficient** **(*SE*)**	***OR***	**[95% CI]**	**Coefficient** **(*SE*)**	***OR***	**[95% CI]**	**Coefficient** **(*SE*)**	***OR***	**[95% CI]**
Age	0.01 (0.02)	1.01	[0.97, 1.04]	−0.03 (0.02)	0.97	[0.94, 1.01]	0.02 (0.02)	1.02	[0.99, 1.06]	−0.00 (0.02)	1.00	[0.96, 1.04]
Sex	−0.39 (0.23)	0.68	[0.43, 1.05]	0.02 (0.23)	1.02	[0.66, 1.59]	0.10 (0.25)	1.10	[0.69, 1.76]	0.69 (0.26) **	2.11	[1.27, 3.52]
Gender identity	−0.21 (0.28)	0.82	[0.47, 1.42]	0.10 (0.29)	1.11	[0.63, 1.94]	−0.66 (0.31) *	0.52	[0.28, 0.96]	0.20 (0.30)	1.23	[0.69, 2.21]
Sexual orientation												
Bisexual/Pansexual	−0.42 (0.24)	0.66	[0.41, 1.06]	−0.29 (0.25)	0.75	[0.46, 1.22]	−0.42 (0.25)	0.65	[0.40, 1.08]	−0.06 (0.27)	0.94	[0.56, 1.58]
Queer/Other	−0.20 (0.38)	0.82	[0.39, 1.73]	0.06 (0.38)	1.06	[0.51, 2.22]	−0.67 (0.42)	0.51	[0.23, 1.16]	−0.35 (0.41)	0.70	[0.31, 1.58]
Race/Ethnicity												
Asian	0.15 (0.37)	1.16	[0.57, 2.39]	−0.21 (0.38)	0.81	[0.38, 1.73]	−0.62 (0.43)	0.54	[0.23, 1.26]	−0.13 (0.44)	0.88	[0.37, 2.07]
Black	0.90 (0.31) **	2.47	[1.35, 4.51]	−0.46 (0.30)	0.63	[0.35, 1.14]	1.07 (0.31) **	2.93	[1.59, 5.40]	0.56 (0.33)	1.75	[0.92, 3.31]
Latine	0.32 (0.31)	1.38	[0.76, 2.51]	0.02 (0.30)	1.02	[0.56, 1.84]	0.75 (0.31) *	2.11	[1.16, 3.85]	0.33 (0.33)	1.40	[0.74, 2.64]
Multi-racial	0.40 (0.57)	1.49	[0.49, 4.51]	−0.02 (0.62)	0.98	[0.29, 3.32]	0.21 (0.63)	1.23	[0.36, 4.22]	0.27 (0.60)	1.31	[0.40, 4.28]
Native American	0.39 (0.55)	1.48	[0.51, 4.35]	−0.61 (0.59)	0.55	[0.17, 1.75]	0.38 (0.65)	1.46	[0.41, 5.24]	−0.18 (0.65)	0.84	[0.23, 3.02]
General life stress	0.17 (0.23)	1.18	[0.75, 1.87]	0.37 (0.24)	1.45	[0.91, 2.31]	−0.40 (0.24)	0.67	[0.42, 1.08]	0.46 (0.26)	1.59	[0.96, 2.64]
Sexual minority stress	0.38 (0.14) **	1.46	[1.10, 1.92]	−0.03 (0.14)	1.03	[0.78, 1.37]	−0.26 (0.15)	0.77	[0.58, 1.03]	0.56 (0.15) ***	1.75	[1.29, 2.37]
Log likelihood	−402.07			−375.28			−346.18			−336.91		
Likelihood ratio *χ*^2^_(12)_	39.84			12.14			41.97			42.77		
Probability > *χ*^2^	0.000			0.434			0.000			0.000		
Pseudo *R*^2^	0.047			0.016			0.057			0.060		
**Independent Variable**	**Reading**				**Creative or Artistic Pursuits**				**Ignore or Avoid**			
	**Coefficient** **(*SE*)**		***OR***	**[95% CI]**	**Coefficient** **(*SE*)**		***OR***	**[95% CI]**	**Coefficient** **(*SE*)**		***OR***	**[95% CI]**
Age	−0.00 (0.02)		1.00	[0.96, 1.04]	−0.30 (0.02)		0.97	[0.94, 1.00]	0.02 (0.02)		1.02	[0.98, 1.06]
Sex	0.32 (0.23)		1.38	[0.88, 2.17]	0.34 (0.23)		1.41	[0.90, 2.20]	−0.09 (0.23)		0.91	[0.58, 1.43]
Gender identity	0.37 (0.29)		1.45	[0.83, 2.53]	0.46 (0.28)		1.58	[0.90, 2.76]	−0.28 (0.28)		0.75	[0.43, 1.32]
Sexual orientation												
Bisexual/Pansexual	0.24 (0.25)		1.28	[0.79, 2.06]	0.14 (0.24)		1.15	[0.71, 1.86]	0.03 (0.25)		1.03	[0.63, 1.66]
Queer/Other	−0.18 (0.38)		0.84	[0.40, 1.77]	−0.24 (0.37)		0.79	[0.38, 1.61]	0.09 (0.37)		1.10	[0.53, 2.26]
Race/Ethnicity												
Asian	−0.67 (0.40)		0.51	[0.23, 1.11]	0.25 (0.38)		1.28	[0.61, 2.69]	0.30 (0.40)		1.34	[0.61, 2.95]
Black	0.37 (0.31)		1.45	[0.79, 2.69]	0.54 (0.30)		1.72	[0.96, 3.09]	−0.10 (0.30)		0.90	[0.50, 1.64]
Latinx	0.27 (0.31)		1.32	[0.72, 2.41]	0.07 (0.31)		1.07	[0.59, 1.95]	−0.34 (0.31)		0.71	[0.50, 1.31]
Multi-racial	−0.07 (0.61)		0.94	[0.28, 3.11]	−0.08 (0.64)		0.92	[0.26, 3.23]	0.19 (0.63)		1.21	[0.39, 4.20]
Native American	−0.06 (0.56)		0.94	[0.31, 2.82]	1.15 (0.61)		3.17	[0.96, 10.49]	0.38 (0.59)		1.46	[0.46, 4.61]
General life stress	0.41 (0.23)		1.51	[0.95, 2.42]	0.59 (0.23) *		1.80	[1.13, 3.36]	0.82 (0.24) **		2.28	[1.42, 3.65]
Sexual minority stress	0.90 (0.15) ***		2.46	[1.83, 3.31]	0.41 (0.14) **		1.50	[1.14, 1.99]	0.25 (0.14)		1.29	[0.97, 1.71]
Log likelihood	−375.60				−398.69				−389.63			
Likelihood ratio *χ*^2^_(12)_	74.59				48.74				27.60			
Probability > *χ*^2^	0.000				0.000				0.006			
Pseudo *R*^2^	0.090				0.058				0.034			

Note. For sex, male was the reference group. For gender identity, cisgender was the reference group. For sexual orientation, gay/lesbian was the reference group. For race, White was the reference group. * *p* < 0.05; ** *p* < 0.01; *** *p* < 0.001.

**Table 4:** Regression analyses predicting anxiety and depressive symptoms.

Independent Variable	Anxiety Symptoms			Depressive Symptoms		
	B (*SE*)	95% CI	β	B *(SE*)	95% CI	β
Age	−0.01 (0.00)	[−0.02, 0.00]	−0.05	−0.01 (0.01)	[−0.02, 0.01]	−0.03
Sex	**0.17 (0.06)** **	[0.06, 0.29]	0.13	0.07 (0.07)	[−0.08, 0.21]	0.04
Gender identity	−0.03 (0.07)	[−0.18, 0.11]	−0.02	0.04 (0.09)	[−0.14, 0.23]	0.02
Sexual orientation						
Bisexual/Pansexual	0.07 (0.06)	[−0.05, 0.19]	0.05	0.25 (0.08) **	[0.10, 0.41]	0.14
Queer/Other	0.15 (0.10)	[−0.04, 0.34]	0.07	0.30 (0.13) *	[0.05, 0.55]	0.10
Race						
Asian	−0.07 (0.10)	[−0.27, 0.13]	−0.03	−0.07 (0.13)	[−0.32, 0.18]	−0.02
Black	−0.11 (0.08)	[−0.27, 0.04]	−0.06	−0.22 (0.10) *	[−0.42, −0.01]	−0.09
Latinx	0.00 (0.08)	[−0.16, 0.15]	0.00	−0.21 (0.10) *	[−0.41, −0.01]	−0.08
Multi-racial	0.04 (0.16)	[−0.27, 0.35]	0.01	0.04 (0.20)	[−0.36, 0.44]	0.01
Native American	0.34 (0.15) *	[0.04, 0.64]	0.09	0.21 (0.20)	[−0.18, 0.60]	0.04
General life stress	0.66 (0.06) ***	[0.54, 0.78]	0.50	1.09 (0.08) ***	[0.93, 1.24]	0.60
Sexual minority stress	0.10 (0.04) *	[0.02, 0.17]	0.12	0.11 (0.05) *	[0.01, 0.21]	0.09
Meditation or mindfulness	−0.04 (0.06)	[−0.13, 0.05]	−0.04	−0.15 (0.06) *	[−0.26, −0.04]	−0.12
Praying, religious/spiritual	0.01 (0.05)	[−0.09, 0.11]	0.01	0.08 (0.06)	[−0.04, 0.21]	0.05
Activism	−0.00 (0.04)	[−0.08, 0.08]	−0.00	−0.09 (0.05)	[−0.19, 0.01]	−0.07
Counseling, psychotherapy, or support group	0.16 (0.04) ***	[0.07, 0.25]	0.15	0.13 (0.06) *	[0.02, 0.24]	0.09
Exercises or physical activities	−0.08 (0.04) *	[−0.16, −0.01]	−0.10	−0.13(0.05) *	[−0.23, −0.03]	−0.11
Talking to friends	0.01 (0.04)	[−0.08, 0.09]	0.00	0.07(0.06)	[−0.04, 0.18]	0.05
Talking to family	0.03 (0.04)	[−0.06, 0.11]	0.03	−0.04(0.06)	[−0.15, 0.07]	−0.03
Journaling	−0.01 (0.04)	[−0.10, 0.08]	−0.01	0.04(0.06)	[−0.08, 0.15]	0.03
Reading	0.00 (0.04)	[−0.08, 0.09]	0.00	−0.02(0.05)	[−0.13, 0.08]	−0.02
Creative or artistic pursuits	0.06 (0.04)	[−0.02, 0.14]	0.08	0.06(0.05)	[−0.04, 0.17]	0.06
Ignore or avoid	−0.00 (0.04)	[−0.07, 0.07]	−0.00	0.07(0.05)	[−0.02, 0.16]	0.06

Note. For sex, male was the reference group. For gender identity, cisgender was the reference group. For sexual orientation, gay/lesbian was the reference group. For race, White was the reference group. * p < 0.05; ** p < 0.01; *** p < 0.001.

## Data Availability

The data presented in this study are available upon request from the corresponding author. The data are not publicly available due to privacy and confidentiality reasons.

## References

[1] Substance Abuse and Mental Health Services Administration Substance Abuse and Mental Health Services Administration (SAMHSA)’s Public Online Data Analysis System (PDAS) [National Survey on Drug Use and Health 2023, 2021]. https://pdas.samhsa.gov/#/survey/NSDUH-2021-DS0001.

[2] Rodriguez-Seijas C., Eaton N.R., Pachankis J.E. (2019). Prevalence of psychiatric disorders at the intersection of race and sexual orientation: Results from the National Epidemiologic Survey of Alcohol and Related Conditions-III. J. Consult. Clin. Psychol..

[3] Meyer I.H. (2003). Prejudice, social stress, and mental health in lesbian, gay, and bisexual populations: Conceptual issues and research evidence. Psychol. Bull..

[4] Brooks V.R. (1981). Minority Stress and Lesbian Women.

[5] Meyer I.H. (2007). Prejudice and discrimination as social stressors. The Health of Sexual Minorities: Public Health Perspectives on Lesbian, Gay, Bisexual and Transgender Populations.

[6] Snyder C.R. (1999). Coping: The Psychology of What Works.

[7] Lazarus R.S. (1966). Psychological Stress and the Coping Process.

[8] Goldbach J.T., Gibbs J.J. (2015). Strategies employed by sexual minority adolescents to cope with minority stress. Psychol. Sex. Orientat. Gend. Divers..

[9] Mulavu M., Anitha Menon J., Mulubwa C., Matenga TF L., Nguyen H., MacDonell K., Wang B., Mweemba O. (2023). Psychosocial challenges and coping strategies among people with minority gender and sexual identities in Zambia: Health promotion and human rights implications. Health Psychol. Behav. Med..

[10] David S., Knight B.G. (2008). Stress and coping among gay men: Age and ethnic differences. Psychol. Aging.

[11] Ngamake S.T., Walch S.E., Raveepatarakul J. (2016). Discrimination and sexual minority mental health: Mediation and moderation effects of coping. Psychol. Sex. Orientat. Gend. Divers..

[12] Lehavot K. (2012). Coping strategies and health in a national sample of sexual minority women. Am. J. Orthopsychiatry.

[13] Hidalgo M.A., Layland E., Kubicek K., Kipke M. (2020). Sexual racism, psychological symptoms, and mindfulness among ethnically/racially diverse young men who have sex with men: A moderation analysis. Mindfulness.

[14] Li M.J., DiStefano A.S., Thing J.P., Black D.S., Simpson K., Unger J.B., Milam J., Contreras R., Bluthenthal R.N. (2019). Seeking refuge in the present moment: A qualitatively refined model of dispositional mindfulness, minority stress, and psychosocial health among Latino/a sexual minorities and their families. Psychol. Sex. Orientat. Gend. Divers..

[15] Lyons A. (2016). Mindfulness attenuates the impact of discrimination on the mental health of middle-aged and older gay men. Psychol. Sex. Orientat. Gend. Divers..

[16] Brewster M.E., Velez B.L., Foster A., Esposito J., Robinson M.A. (2016). Minority stress and the moderating role of religious coping among religious and spiritual sexual minority individuals. J. Couns. Psychol..

[17] Lauricella S.K., Phillips R.E., Dubow E.F. (2017). Religious coping with sexual stigma in young adults with same-sex attractions. J. Relig. Health.

[18] Porter K.E., Brennan-Ing M., Burr J.A., Dugan E., Karpiak S.E. (2019). HIV stigma and older men’s psychological well-being: Do coping resources differ for gay/bisexual and straight men?. J. Gerontol. Ser. B.

[19] Ehlke S.J., Braitman A.L., Dawson C.A., Heron K.E., Lewis R.J. (2020). Sexual minority stress and social support explain the association between sexual identity with physical and mental health problems among young lesbian and bisexual women. Sex Roles.

[20] Fingerhut A.W. (2018). The role of social support and gay identity in the stress processes of a sample of Caucasian gay men. Psychol. Sex. Orientat. Gend. Divers..

[21] Hambour V.K., Duffy A.L., Zimmer-Gembeck M.J. (2023). Social identification dimensions, sources of discrimination, and sexuality support as correlates of well-being among sexual minorities. J. Appl. Soc. Psychol..

[22] Wang Y., Lao C.K., Wang Q., Zhou G. (2022). The impact of sexual minority stigma on depression: The roles of resilience and family support. Sex. Res. Soc. Policy.

[23] Lewis R.J., Derlega V.J., Clarke E.G., Kuang J.C., Jacobs A.M., McElligott M.D. (2005). An expressive writing intervention to cope with lesbian-related stress: The moderating effects of openness about sexual orientation. Psychol. Women Q..

[24] Pachankis J.E., Williams S.L., Behari K., Job S., McConocha E.M., Chaudoir S.R. (2020). Brief online interventions for LGBTQ young adult mental and behavioral health: A randomized controlled trial in a high-stigma, low-resource context. J. Consult. Clin. Psychol..

[25] Dajches L., Aubrey J.S. (2022). Queer folklore: Examining the influence of fandom on sexual identity development and fluidity acceptance among Taylor Swift fans. Psychol. Pop. Media.

[26] Bergfeld J.R., Chiu E.Y. (2017). Mediators in the relationship between minority stress and depression among young same-sex attracted women. Prof. Psychol. Res. Pract..

[27] Montagno M.J., Garrett-Walker J.J. (2022). LGBTQ+ engagement in activism: An examination of internalized heterosexism and LGBTQ+ community connectedness. J. Homosex..

[28] Sattler F.A., Wagner U., Christiansen H. (2016). Effects of minority stress, group-level coping, and social support on mental health of German gay men. PLoS ONE.

[29] Pharr J.R., Flatt J.D., Chien L.-C., Kachen A., Olakunde B.O. (2021). Exercise as a mitigator of poor mental health among lesbian, gay, and bisexual adults. J. Phys. Act. Health.

[30] Craig S.L., Eaton A.D., Leung VW Y., Iacono G., Pang N., Dillon F., Austin A., Pascoe R., Dobinson C. (2021). Efficacy of affirmative cognitive behavioural group therapy for sexual and gender minority adolescents and young adults in community settings in Ontario, Canada. BMC Psychol..

[31] Pachankis J.E., Hatzenbuehler M.L., Rendina H.J., Safren S.A., Parsons J.T. (2015). LGB-affirmative cognitive-behavioral therapy for young adult gay and bisexual men: A randomized controlled trial of a transdiagnostic minority stress approach. J. Consult. Clin. Psychol..

[32] Pachankis J.E., McConocha E.M., Clark K.A., Wang K., Behari K., Fetzner B.K., Brisbin C.D., Scheer J.R., Lehavot K. (2020). A transdiagnostic minority stress intervention for gender diverse sexual minority women’s depression, anxiety, and unhealthy alcohol use: A randomized controlled trial. J. Consult. Clin. Psychol..

[33] Litman L., Robinson J., Abberbock T. (2017). Turkprime.com: A versatile crowdsourcing data acquisition platform for the Behavioral Sciences. Behav. Res. Methods.

[34] Buhrmester M., Kwang T., Gosling S.D. (2016). Amazon’s Mechanical Turk: A new source of inexpensive, yet high-quality, data?. Perspect. Psychol. Sci..

[35] Chandler J., Rosenzweig C., Moss A.J., Robinson J., Litman L. (2019). Online panels in social science research: Expanding Sampling Methods Beyond Mechanical Turk. Behav. Res. Methods.

[36] Coppock A. (2019). Generalizing from survey experiments conducted on mechanical turk: A replication approach. Political Sci. Res. Methods.

[37] Krupnikov Y., Levine A.S. (2014). Cross-sample comparisons and external validity. J. Exp. Political Sci..

[38] Levay K.E., Freese J., Druckman J.N. (2016). The demographic and political composition of Mechanical Turk samples. SAGE Open.

[39] Mortensen K., Hughes T.L. (2018). Comparing Amazon’s mechanical turk platform to conventional data collection methods in the Health and Medical Research Literature. J. Gen. Intern. Med..

[40] Mullinix K.J., Leeper T.J., Druckman J.N., Freese J. (2015). The generalizability of survey experiments. J. Exp. Political Sci..

[41] Shapiro D.N., Chandler J., Mueller P.A. (2013). Using mechanical turk to study clinical populations. Clin. Psychol. Sci..

[42] Beck A.T., Epstein N., Brown G., Steer R.A. (1988). An inventory for measuring clinical anxiety: Psychometric properties. J. Consult. Clin. Psychol..

[43] Zimmerman M., Chelminski I., McGlinchey J.B., Posternak M.A. (2008). A clinically useful depression outcome scale. Compr. Psychiatry.

[44] Kohn P.M., Macdonald J.E. (1992). The Survey of Recent Life Experiences: A decontaminated hassles scale for adults. J. Behav. Med..

[45] Frey J.J. (2020). Multidimensional Stigma and the Mental Health of Sexual Minority Adults. Doctoral Dissertation.

[46] Little R., Rubin D. (2019). Statistical Analysis with Missing Data, Third Edition.

[47] McLachlan G.J., Krishnan T. (2008). The EM Algorithm and Extensions.

[48] Hsieh F.Y. (1989). Sample size tables for logistic regression. Stat. Med..

[49] Bujang M.A., Sa’at N., Sidik TM IT A.B., Joo L.C. (2018). Sample Size Guidelines for Logistic Regression from Observational Studies with Large Population: Emphasis on the Accuracy between Statistics and Parameters Based on Real Life Clinical Data. Malays. J. Med. Sci. MJMS.

[50] Gonzalez K.A., Pulice-Farrow L., Abreu R.L. (2022). “In the voices of people like me”: LGBTQ coping during Trump’s administration. Couns. Psychol..

[51] Frost D.M., Meyer I.H., Schwartz S. (2016). Social support networks among diverse sexual minority populations. Am. J. Orthopsychiatry.

[52] Diamond L.M., Alley J. (2022). Rethinking minority stress: A social safety perspective on the health effects of stigma in sexually-diverse and gender-diverse populations. Neurosci. Biobehav. Rev..

[53] Szymanski D.M., Owens G.P. (2008). Do coping styles moderate or mediate the relationship between internalized heterosexism and sexual minority women’s psychological distress?. Psychol. Women Q..

[54] Szymanski D.M., Dunn T.L., Ikizler A.S. (2014). Multiple minority stressors and psychological distress among sexual minority women: The roles of rumination and maladaptive coping. Psychol. Sex. Orientat. Gend. Divers..

[55] Szymanski D.M., Kashubeck-West S., Meyer J. (2008). Internalized heterosexism: Measurement, psychosocial correlates, and research directions. Couns. Psychol..

[56] Woodell B., Schwadel P. (2020). Changes in religiosity among lesbian, gay, and bisexual emerging adults. J. Sci. Study Relig..

[57] Conron K.J., Goldberg S.K., O’Neill K.K. (2020). Religiosity among LGBT Adults in the US.

[58] Dunbar M.S., Sontag-Padilla L., Ramchand R., Seelam R., Stein B.D. (2017). Mental health service utilization among lesbian, gay, bisexual and questioning or queer college students. J. Adolesc. Health.

[59] Rees S.N., Crowe M., Harris S. (2021). The lesbian, gay, bisexual and transgender communities’ mental health care needs and experiences of mental health services: An integrative review of qualitative studies. J. Psychiatr. Ment. Health Nurs..

[60] Silveri G., Schimmenti S., Prina E., Gios L., Mirandola M., Converti M., Fiorillo A., Pinna F., Ventriglio A., Galeazzi G.M. (2022). Barriers in care pathways and unmet mental health needs in LGBTIQ + communities. Int. Rev. Psychiatry.

[61] Brougham R.R., Zail C.M., Mendoza C.M., Miller J.R. (2009). Stress, sex differences, and coping strategies among college students. Curr. Psychol..

[62] Meléndez J.C., Mayordomo T., Sancho P., Tomás J.M. (2012). Coping strategies: Gender differences and development throughout life span. Span. J. Psychol..

[63] Tamres L.K., Janicki D., Helgeson V.S. (2002). Sex differences in coping behavior: A meta-analytic review and an examination of relative coping. Personal. Soc. Psychol. Rev..

[64] Range L.M., Jenkins S.R. (2010). Who benefits from Pennebaker’s expressive writing paradigm? Research recommendations from three gender theories. Sex Roles.

[65] Budge S.L., Katz-Wise S.L., Tebbe E.N., Howard KA S., Schneider C.L., Rodriguez A. (2013). Transgender emotional and coping processes: Facilitative and avoidant coping throughout gender transitioning. Couns. Psychol..

[66] Koken J.A., Bimbi D.S., Parsons J.T. (2009). Experiences of familial acceptance–rejection among transwomen of color. J. Fam. Psychol..

[67] von Doussa H., Power J., Riggs D.W. (2020). Family matters: Transgender and gender diverse peoples’ experience with family when they transition. J. Fam. Stud..

[68] Gray A., Desmarais S. (2014). Not all one and the same: Sexual identity, activism, and collective self-esteem. Can. J. Hum. Sex..

[69] Rollins J., Hirsch H.N. (2003). Sexual identities and political engagements: A queer survey. Soc. Politics Int. Stud. Gend. State Soc..

[70] Galinsky A.D., Wang C.S., Whitson J.A., Anicich E.M., Hugenberg K., Bodenhausen G.V. (2013). The Reappropriation of Stigmatizing Labels: The Reciprocal Relationship between Power and Self-Labeling. Psychol. Sci..

[71] Worthen MG F. (2023). Queer identities in the 21st century: Reclamation and stigma. Curr. Opin. Psychol..

[72] Kane L., Benson K., Stewart Z.J., Daughters S.B. (2024). The impact of spiritual well-being and social support on substance use treatment outcomes within a sample of predominantly Black/African American adults. J. Subst. Use Addict. Treat..

[73] Blank M.B., Mahmood M., Fox J.C., Guterbock T. (2002). Alternative mental health services: The role of the black church in the South. Am. J. Public Health.

[74] Green D.S., Chuang S.S. (2023). Impacts of religion on established adult women’s lives and development: Black Jamaican women’s perspectives. J. Adult Dev..

[75] Watson-Singleton N.N., Black A.R., Spivey B.N. (2019). Recommendations for a culturally-responsive mindfulness-based intervention for African Americans. Complement. Ther. Clin. Pract..

[76] Woods-Giscombé C.L., Gaylord S.A. (2014). The cultural relevance of mindfulness meditation as a health intervention for African Americans: Implications for reducing stress-related health disparities. J. Holist. Nurs..

[77] Proulx J., Croff R., Hebert M., Oken B. (2020). Results of a mindfulness intervention feasibility study among elder African American women: A qualitative analysis. Complement. Ther. Med..

[78] Foster K.A., Bowland S.E., Vosler A.N. (2015). All the pain along with all the joy: Spiritual resilience in lesbian and gay Christians. Am. J. Community Psychol..

[79] Gandy M.E., Natale A.P., Levy D.L. (2021). “We shared a heartbeat”: Protective functions of faith communities in the lives of LGBTQ+ people. Spiritual. Clin. Pract..

[80] Belcher B.R., Berrigan D., Dodd K.W., Emken B.A., Chou C.P., Spruijt-Metz D. (2010). Physical activity in US youth: Effect of race/ethnicity, age, gender, and weight status. Med. Sci. Sports Exerc..

[81] Ellis K.R., Griffith D.M., Allen J.O., Thorpe Jr R.J., Bruce M.A. (2015). “If you do nothing about stress, the next thing you know, you’re shattered”: Perspectives on African American men’s stress, coping and health from African American men and key women in their lives. Soc. Sci. Med..

[82] Crockett L.J., Iturbide M.I., Torres Stone R.A., McGinley M., Raffaelli M., Carlo G. (2007). Acculturative stress, social support, and coping: Relations to psychological adjustment among Mexican American college students. Cult. Divers. Ethn. Minor. Psychol..

[83] Abate A., Bailey C., Venta A. (2022). Attachment and Social Support in Latinx Young Adults: Investigating the Moderating Role of familismo. J. Cross-Cult. Psychol..

[84] Campos B., Ullman J.B., Aguilera A., Dunkel Schetter C. (2014). Familism and psychological health: The intervening role of closeness and social support. Cult. Divers. Ethn. Minor. Psychol..

[85] Collins K.M., Levitt H.M., Maroney M.R. (2020). Peeling Back the Layers: How Expressive Writing about Heterosexist Events Benefits Sexual Minority Adults. J. Homosex..

[86] Levitt H.M. (2022). Applications of a Functionalist Theory of Gender: A Response to Reflections and a Research Agenda. Psychol. Women Q..

[87] Lucassen MF G., Núñez-García A., Rimes K.A., Wallace L.M., Brown K.E., Samra R. (2022). Coping strategies to enhance the mental wellbeing of sexual and gender minority youths: A scoping review. Int. J. Environ. Res. Public Health.

[88] Toomey R.B., Ryan C., Diaz R.M., Russell S.T. (2018). Coping with sexual orientation related minority stress. J. Homosex..

[89] Anderson E., Shivakumar G. (2013). Effects of exercise and physical activity on anxiety. Front. Psychiatry.

[90] Keng S.L., Smoski M.J., Robins C.J. (2011). Effects of mindfulness on psychological health: A review of empirical studies. Clin. Psychol. Rev..

[91] Burton C.L., Wang K., Pachankis J.E. (2019). Psychotherapy for the spectrum of sexual minority stress: Application and technique of the ESTEEM treatment model. Cogn. Behav. Pract..

[92] Hall W.J., Ruiz Rosado B., Chapman M.V. (2019). Findings from a feasibility study of an adapted cognitive behavioral therapy group intervention to reduce depression among LGBTQ (lesbian, gay, bisexual, transgender, or queer) young people. J. Clin. Med..

[93] Jenkins W.D., Walters S., Phillips G., Green K., Fenner E., Bolinski R., Spenner A., Luckey G. (2024). Stigma, Mental Health, and Health care Use Among Rural Sexual and Gender Minority Individuals. Health Educ. Behav..

[94] Hatzenbuehler M.L., Nolen-Hoeksema S., Dovidio J. (2009). How does stigma “get under the skin”? The mediating role of emotion regulation. Psychol. Sci..

[95] Craig S.L., McInroy L.B., Eaton A.D., Iacono G., Leung V.W., Austin A., Dobinson C. (2019). An affirmative coping skills intervention to improve the mental and sexual health of sexual and gender minority youth (project youth AFFIRM): Protocol for an implementation study. JMIR Res. Protoc..

[96] McDermott E., Eastham R., Hughes E., Pattinson E., Johnson K., Davis S., Pryjmachuk S., Mateus C., Jenzen O. (2021). Explaining effective mental health support for LGBTQ+ youth: A meta-narrative review. SSM-Ment. Health.

[97] Della Casa V., Gubello A., Malmquist A., Mezzalira S., Bonato M., Simonelli A., Gatta M., Miscioscia M. (2024). Minority stress, self-awareness, and coping strategies during the COVID-19 pandemic among Italian transgender young adults. Healthcare.

[98] Wilson C., Cariola L.A. (2020). LGBTQI+ youth and mental mealth: A systematic review of qualitative research. Adolesc. Res. Rev..

